# Application Value of Serum Multi-Antibody Combined Detection in Differential Diagnosis and Typing of Lung Cancer

**DOI:** 10.1155/2022/8944263

**Published:** 2022-01-28

**Authors:** Tian Cai, Weishen Yao, Fuyou Liang, Qingshui Yang, Fulang Han

**Affiliations:** Nanhai District People's Hospital of Foshan, Foshan, Guangdong, China

## Abstract

One of the most prevalent malignant tumours is lung cancer. Circulating microRNAs (miRNAs) have shown to have significant promise for lung cancer diagnosis and prognosis, according to a growing body of research. The researchers wanted to explore if serum exosomal miR-1246 has any treatment significance in patients with non-small-cell lung cancer (NON-SCLC). Real-time PCR was used to determine the stage of exosomal miR-1246 serum expression in NON-SCLC patients. The researchers next looked into the link regarding exosomal miR-1246 serum stages and NON-SCLC prognosis. In NON-SCLC patients, exosomal miR-1246 serum appearance was considerably higher. According to a receiver operating characteristic (ROC) research, serum exosomal miR-1246 was effective in discriminating NON-SCLC patients from normal controls and non-malignant respiratory illness patients. Following treatment, the amount of serum exosomal miR-1246 reduced but increased in cases of recurrence. Furthermore, the level of serum exosomal miR-1246 was connected to distant metastases and TNM stages in a significant way. According to a survival analysis, cases with severe levels of exosomal miR-1246 serum had reduced overall or disease-free survival. The level of exosomal miR-1246 serum was found to be an autonomous predictive issue for NON-SCLC in multi-variate analysis. Finally, exosomal miR-1246 serum may be a useful prognosis biomarker for non-small-cell lung cancer.

## 1. Introduction

The most prevalent cancer-related cause of death is lung cancer. China is in the midst of a lung cancer epidemic on an unprecedented scale. In 2015, there were an estimated 733,000 new lung cancer cases (17% of total cancer incidence) and 610,000 deaths (21.7% of total cancer mortality) in China. In 2018, there were roughly 2,093,876 cases of announced lung infections perceived globally with 1,761,007 passings. In 2018, there were roughly 2,093,876 cases of announced lung infections perceived globally with 1,761,007 passings. NON-SCLC breakdown in the lungs is the most continuous sort of cellular breakdown in the lungs, representing generally 85% of all cases. Lung cancer cells have a proclivity for infiltrating surrounding tissue and spreading to distant organs, resulting in disease development, and NON-SCLC patients have a dismal clinical prognosis. Despite substantial advances in medicine, like surgeries, radiation, chemotherapeutics, and laser surgery, the 5-year survival rate for advanced-stage NON-SCLC remains exceedingly low. As a result, it is critical to look into new biomarkers for NON-SCLC early diagnosis and prognosis. MicroRNAs (miRNAs) are a group of non-RNAs that is highly conserved, short, and small (18–24 nucleotides) and serves a critical role in the regulation of mRNA translation and degradation via complementary binding with the 3′-untranslated regions (UTRs) of target genes. Previous research has found significant evidence that over 1500 miRNAs may alter the expression of more than 60% of human genes. Many physiologic processes, like proliferation, migration, invasion, differentiation, and apoptosis, have been discovered to involve miRNAs. Anomaly miRNA expression has also been associated to a number of diseases, particularly cancer. Depending on the targeted genes and tumour microenvironment, these small compounds might serve as oncogenes or tumour suppressors.

Exosomes are tiny vesicles with a diameter of 30–150 nm. Proteins, short-chain peptides, lipids, mRNAs, and non-coding RNAs are among the items they transport. Because the double-lipid layer protects MiRNAs, they are particularly prevalent in exosomes. The abnormal expression of circulating exosomal miRNAs has been shown to be a useful diagnostic for NON-SCLC diagnosis and prognosis. For example, in NON-SCLC, exosomal plasma miR-21 (5p) miR-10b (5p), and miR-23b (3p) expression levels were raised, and overexpression of these plasma exosomal miRNAs was linked to a poor prognosis. miR-1246 has been demonstrated to be important in the onset and development of NON-SCLC. However, it was unclear whether serum exosomal miR-1246 is overexpressed in NON-SCLC, and if so, it was not clear what clinical importance it would have. As a result, the purpose of this study was to see if serum exosomal miR-1246 had any prognostic and predictive value in NON-SCLC.

## 2. Related Works

Lung cancer became more common among past smoking than cigarette smokers in the USA [1]. However, in other countries, such as China, where smoking prevalence has climbed substantially over the previous couple of decades, lung cancer prevalence is still expected to reach high with a high of every 2nd adult male in China smokes accounting with one of all smokes worldwide. In the USA, NON-SCLC accounted for 88% of all cases of lung cancer. Following a positive diagnosis, proper non-small-cell lung cancer grading with computerized tomography and positron emission is essential for determining the best treatment options. Herbst et al. [2] clarify significant progressions in the administration of NON-SCLC non little cell cellular breakdown in the lungs have been made in the past twnety years, remembering propels for malignancy science and cancer arrangement pathways, just as early location and multimodal care. Little atom tyrosine kinase inhibitors and immunotherapy have brought about remarkable endurance expansions in certain patients. In [3], 1207 grown-up patients non-little cell cellular breakdown in the lungs being relegated to one of three investigational medicines: cisplatin and gemcitabine, cisplatin and docetaxel, or carboplatin and paclitaxel. The headway of cellular breakdown in the lungs organizing toward non-intrusive, endoscopy-based, and illustrations draws near, just as the foundation of stage-adjusted treatment, is talked about in [4]. Patients with metastatic disease and NON-SCLC were investigated in [5] where nivolumab + ipilimumab serum was taken at three time points: baseline, 2–4 weeks after the first dose, and at the uses the opportunity at the time points. Sandwich ELISA was used to measure the levels of IL-8 in the blood. Because of its minimum invasive, easy, and well-accepted features, cell-free circulating tumour DNA (ctDNA) has gotten a lot of attention as a potential tumour marker [6]. NON-SCLC patients had significantly more ctDNA in their plasma or serum than healthy controls or patients with benign illnesses. Besides, a connection was established between growth stage, cancer grade, lymph hub contribution, quantity of metastatic locales, cancer reaction, endurance result, and ctDNA levels in various examinations [7]. Understanding the roles of microRNAs (miRNAs) in carcinogenesis and their value as cancer biomarkers has been a focus of recent research. miR-21 was studied as a possible blood biomarker for NON-SCLC in this study. Real-time PCR was used to sense the relative appearance stage of miR21 in the sera of 80 NON-SCLC patients as well as 60 healthy people (control group). It was determined via a ROC curve and the clinical outcome of serum miR-21 concentrations. The VEGF, essential fibroblast development factor (bFGF), endostatin, and thrombospondin 1 (TSP1) plasma and serum focuses were inspected in 21 advanced nonlittle cell cellular breakdown in the lungs (SCLC) victims and 46 solid control individuals in [8]. We additionally checked out the connection between these levels and the result of the condition. The protein connected immunosorbent examines were utilized to recognize cytokine levels in plasma and serum at three focuses: before treatment, multi-week subsequent to beginning chemotherapy, and 12 weeks in the wake of completing chemotherapy. In [9], we started a longitudinal prospective trial in a Chinese PLA general hospital on advanced NON-SCLC patients treated with PD-1/PD-L1 inhibitors (Beijing, China). Blood samples were taken at the beginning and at the end of the 6-week therapy period. All patients underwent a CT scan to assess therapy efficacy in accordance with RECIST. SCC-Ag levels were determined by electrochemical luminescence, while serum CEA, CA125, and CYFRA21-1 levels were determined using a chemiluminescent microparticle immunoassay. Reference [10], for instance, The objective of this review was to discover serum microRNAs (miRNAs) connected to the TGF flagging pathway as indicators of endurance in cutting edge non-little cell cellular breakdown in the lungs patients (NON-SCLC). Reference [11] is a model. The focal point of this examination was to perceive how serum carcino-undeveloped antigen (CEA) changed and what it implied in patients with privately progressed non-little cell cellular breakdown in the lungs during gefitinib treatment (NON-SCLC). Forty patients with cutting edge NON-SCLC in stages III-IV were picked as study subjects and were treated with gefitinib in mix with standard neighborhood radiotherapy until cancer movement or serious harmfulness. The objective of this review was to check whether recognizing EGFR changes in fringe blood was plausible and valuable for forecast [12]. The amplification refractory mutation system was used to test plasma, serum, and tumour tissue samples from 164 NON-SCLC patients for EGFR mutations (ARMS). The objective of the study was to check out the degrees of miR-19a articulation in non-little cell cellular breakdown in the lung (NON-SCLC) tissue and serum, just as the connections between serum miR-19a articulation and clinical factors and NON-SCLC patient anticipation [13]. An aggregate of 138 progressed NON-SCLC patients and twenty solid controls were remembered for the review [14]. C-responsive protein (CRP) and egg whites were joined to compute GPS. By utilizing a compound connected immunosorbent test, the analysts had the option to recognize three serum growth markers: cytokeratin 19 piece antigen 21-1 (CYFRA21-1), carcinoembryonic antigen (CEA), and tissue polypeptide explicit antigen (TPS) (ELISA). Preceding chemotherapy, GPS and growth markers were assessed. Somewhere around two rounds of cisplatin-based chemotherapy were given to the patients as a whole. Somewhere around two rounds of cisplatin-based chemotherapy were given to the patients as a whole, and following that, there was a 2-to 5-year take on [15]. For the initial 40 patients, the criteria included stage IIIB/IV or recurrent non-squamous, non-small-cell lung cancer (NON-SCLC), no prior treatment for metastatic illness, PS = 0–1, and no history of brain metastases. An additional ten patients were included to the cohort, allowing for the treatment of brain metastases. Patients were given 150 mg of erlotinib each day and 15 mg of bevacizumab per kilogram of body weight every three weeks until objective or clinical development.

## 3. Methods

### 3.1. Serum Specimens and Patients

The study included 105 patients with NON-SCLC, 50 individuals with non-malignant respiratory diseases (NMRDs), and 50 healthy volunteers from Nanjing Medical University, China. Patients who had had any type of anticancer treatment before being diagnosed with NON-SCLC were excluded from the study. Two separate pathologists confirmed all of the NON-SCLC cases pathologically. NON-SCLC was classified using the American Joint Committee on Cancer (AJCC) staging approach.  Table 1 summarizes all clinical data gathered retrospectively from medical records. Patients with NMRD had asthma in 15 cases and bronchiectasis in 21 cases, and 14 people had chronic obstructive pulmonary disease (COPD). The Independent Commission of Harbin Medicine Institution's Second Affiliated Hospitals approved this study, and it was carried out in accordance with the Declaration of Helsinki. All of the participants signed an approval form. All of participants' blood samples were taken and extractor for 15 minutes at 3000 g. The serum solution was encrypted and recorded at −80°C for subsequent use.

### 3.2. qRT-PCR

The manufacturer's mirVanaTM miRNA Isolation System was used to isolate total sum of RNA using sera Samples are USA, Austin, TX, ambion inc. The content and purity of total RNA were determined using a NanoDrop 2000. The PrimeScript RT Reagent Kit was used to convert total RNA to cDNA. cDNA was amplified using the miScript SYBR-Green PCR Kit and the ABI 7500 Real-Time PCR System (Applied Biosystems, Foster, CA, USA). It was done by maintaining 95 degree C for 5 minutes, then for 41 cycles, 94 degree C for 15 sec was maintained, and then 55 degree C for 30 seconds was maintained. A synthesized sudden increase control RNA was created using the Caenorhabditis worms microrna cel-miR-39. Using the 2-Ct method of relative quantification, the appearance of exosomal miR-1246 serum in various methods was analyzed.

### 3.3. Enzyme-Linked Immunosorbent Assay

The Human Carcinoembryonic Antigen ELISA Kit was used to assess the expression of carcinoembryonic antigen (CEA) in NON-SCLC patients and healthy persons (Abcam, Cambridge, MA, USA).

### 3.4. Analytical Statistics

GraphPad version six was used for all surveys and studies (GraphPad Software Inc., San Diego, CA, USA). The exosomal miR-1246 serums levels in various categories were evaluated using the Mann–Whitney *U* test or the Kruskal–Wallis experiment. The chi-square strategy was applied to see if there were any associations among serum exosomal miR-1246 production and clinicopathologic outcomes. The diagnostic performance of blood exosomal miR-1246 was analyzed using ROC. ROC stands for receiver operating curves and the coefficient of determination (accuracy). The survival curve was created by Kaplan–Meier technology and rank-log testing. For multi-variate analysis, the Cox regression model is applied. The significance threshold was set at *P* < 0.05.

### 3.5. Exosomal Serum miR-1246 Is Boosted Substantially in NON-SCLC

**Figure 1:**
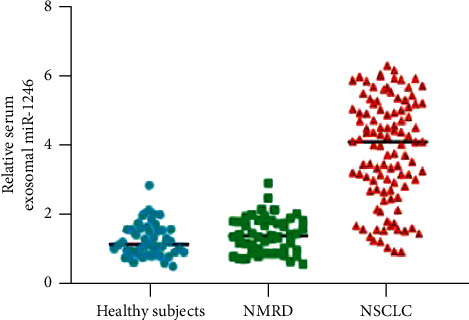
The graphics representation of NON-SCLC, Serum Exosomal miR-1246 production, was significantly greater than in patients with NMRD and healthy participants.

The relative quantities of plasma exosomal miR-1246 for patients with NON-SCLC, participants with NMRD, and healthy controls were determined using qRT-PCR as shown in Figure 1. Exosomal miR-12346 serum appearance in patients with NON-SCLC was substantially higher than that in participants with NMRD and healthy controls (*P* < 0.001).

**Figure 2:**
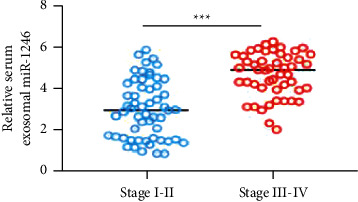
Exosomal miR-1246 levels in the blood were greater in patients with advanced NON-SCLC.

**Figure 3:**
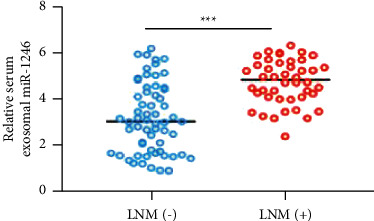
In NON-SCLC patients with higher lymph node metastases, serum exosomal miR-1246 was greater (LNM).

In addition, as compared to their respective controls (*P* < 0.001), NON-SCLC patients with advanced clinical stages or lymph node metastasis exhibited greater levels of serum exosomal miR-1246 (Figures 2 and 3).

### 3.6. Diagnostic Signifiance of Early Stage NON-SCLC, Serum Exosomal miR-1246

**Figure 4:**
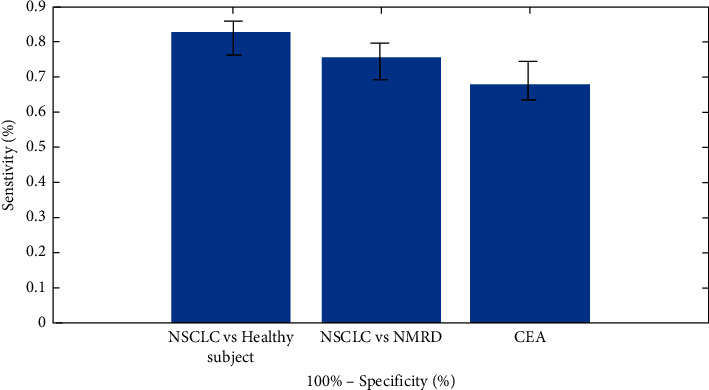
Exosomal miR-1246 in serum has diagnostic significance for NON-SCLC.

There were 33 NON-SCLC cases in total, all of which were in stage I. The diagnostic utility of serum exosomal miR-1246 towards early detection of NON-SCLC was examined. According to ROC curve, serum exosomal miR-1246 discriminated early-stage NON-SCLC patients from healthy people with an accuracy of 0.839. The accuracy value of serum exosomal miR-1246, which distinguished individuals with early-stage NON-SCLC from those with NMRD, was 0.757. The accuracy value of the classic tumour marker CEA in discriminating initial stage NON-SCLC patients and normal control is depicted in Figure 4.

### 3.7. Serum Exosomal miR-1246 Decreased following Treatments and Increased with Recurrence

**Figure 5:**
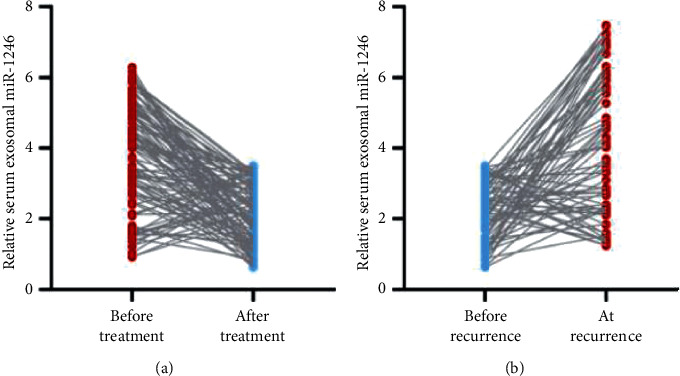
Link between exosomal miR-1246 in the blood and therapeutic responsiveness and recurrence.

Before and after treatments, the quantities of serum exosomal miR-1246 transcription were investigated. Following treatment, the serum exosomal miR-1246 level appeared to be strongly reduced (*P* < 0.001). Recurrence occurred in 74 cases, and serum exosomal miR-1246 levels are significantly enhanced (*P* < 0.001) at the time of recurrence, as shown in Figures 5(a) and 5(b).

### 3.8. Clinical Outcome of Exosomal miR-1246 in Serum and NON-SCLC

**Figure 6:**
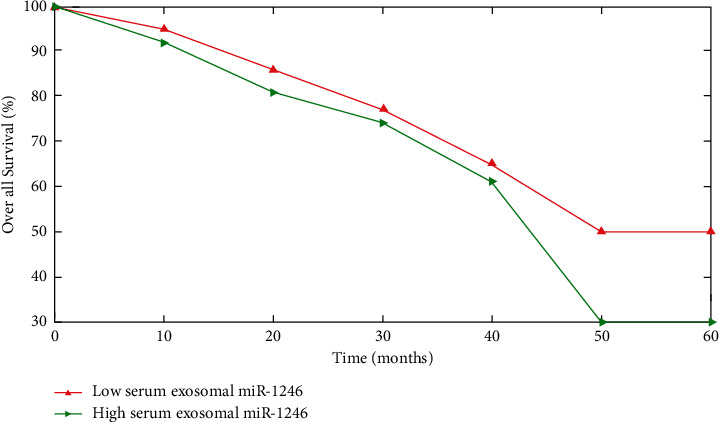
Higher serum exosomal miR-1246 with NON-SCLC cases.

**Figure 7:**
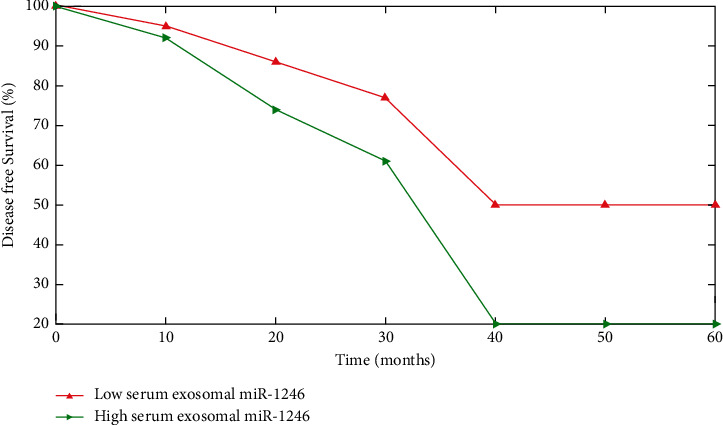
Patients with NON-SCLC who had a high level of exosomal miR-1246 in their blood had a shorter disease-free survival than those who had a low level of exosomal miR-1246 in their blood.

All NON-SCLC samples were divided into two groups: those with high serum exosomal miR-1246 levels and those with decreased plasma exosomal miR-1246 levels, depending on the average mean serum exosomal miR-1246 in NON-SCLC patients. Progressive TNM phase (*P* < 0.001) and lymph node metastases (*P* < 0.001) and increased serum cells and tissue miR-1246 expressions were positively related (Figures 6 and 7).

The increased serum exosomal miR-1246 group exhibited worse survival rates (OS) (*P*=0.0002) or illness survival (*P* < 0.0001) than that of the decreased serum exosomal miR-1246 category (Figures 6 and 7). The multi-variate analysis revealed that TNM stage (*P* < 0.001), distant metastases (*P*=0.007) and exosomal miR-1246 serum (*P*=0.012) are all independent predictors of OS.

## 4. Discussion

The importance of early detection and prognosis in NON-SCLC is critical for lowering mortality. Unfortunately, there is currently no good biomarker for SCLC. The serum exosomal miR-1246 was found to be considerably enhanced in individuals with NON-SCLC in the current investigation. Furthermore, serum exosomal miR-1246 upregulation distinguished early-stage NON-SCLC from healthy individuals and patients with NMRD. Following treatments, serum exosomal miR-1246 levels reduced but increased in cases of recurrence. Furthermore, elevated serum exosomal miR-1246 was found to be associated with aggressive clinic pathologic measures as well as poor OS/RFS. Exosomal miR-1246 was found to be an independent risk factor for NON-SCLC in a multi-variate analysis. These data suggest that serum exosomal miR-1246 could be used as a biomarker for NON-SCLC detection and prognosis [13].

The role of miR-1246 in NON-SCLC has been investigated in several research studies. Knockdown of miR-1246, for example, suppressed the expression of stemness and epithelial-mesenchymal transition markers in NON-SCLC. Furthermore, inhibiting miR-1246 inhibited NON-SCLC cell proliferation, sphere formation, colony formation, and invasion, showing that miR-1246 plays an oncogenic function in NON-SCLC carcinogenesis. The expression of circulating miR-1246 was shown to be higher in patients with NON-SCLC, according to [7]. Lung cancer cells migrated and invaded better when miR-1246 was pushed to be expressed. miR-1246 appears to be associated to lung cancer cells' radioresistance. Following irradiation, both intracellular and extracellular miR-1246 levels rose, and enhanced miR-1246 was substantially linked with radioresistance in NON-SCLC cells. Targeting miR-1246 encouraged lung cancer cell proliferation and increased radioresistance, suggesting that targeting miR-1246 could be a viable technique for improving NON-SCLC therapeutic outcomes.

miR-1246 has also been found to increase tumour growth in other cancers [4]. By blocking thrombospondins, miR-1246 increased cervical cancer cell proliferation, migration, and invasion. Furthermore, when comparing cervical squamous-cell carcinoma patients with lymph node metastases to those without lymph node metastasis, serum miR-1246 expression was considerably elevated. Similarly, miR-1246 expression was shown to be quite high in colorectal cancer (CRC) tissues and cell lines. In CRC patients, serum exosomal miR-1246 levels were also shown to be higher than in healthy controls. After surgical therapy, the amount of serum exosomal miR-1246 was dramatically reduced. As a result, serum exosomal miR-1246 overexpression is not limited to NON-SCLC. Early diagnosis and prognosis will be improved by combining serum exosomal miR-1246 with standard tumour biomarkers as well as clinicopathologic data.

In cancer, miR-1246 may operate as a tumour suppressor. miR-1246 was found to be downregulated in lung cancer cell lines. In vitro, ectopic expression of miR-1246 inhibited lung cancer cell invasion and epithelial-mesenchymal transition, suggesting that miR-1246 may play a tumour suppressive role in lung cancer [14]. These discoveries need to be looked into further. It is probable that miR-1246 plays a varied role at different stages of lung cancer. Furthermore, the findings in vitro may not reflect the real conditions in vivo.

## 5. Conclusions

The significance of serum exosomal miR-1246 in patients with non-small-cell lung cancer (NON-SCLC) was explored in this paper. Real-time PCR was used to determine the stage of exosomal miR-1246 serum expression in NON-SCLC patients. The findings for the first time show that serum exosomal miR-1246 levels are considerably higher in patients with NON-SCLC. Furthermore, increase of serum exosomal miR-1246 is linked to decreased survival and a poor prognosis in NON-SCLC patients. Exosomal miR-1246 was found to be an independent risk factor for NON-SCLC in a multi-variate analysis. These data suggest that serum exosomal miR-1246 could be used as a biomarker for NON-SCLC diagnosis and prognosis. As a result, serum exosomal miR-1246 has the potential to be a useful biomarker for early identification and prognosis.

## Figures and Tables

**Table 1:** Medical records for all clinical data.

Finding	Quantity	Exosomal miR-1246	
		High	Low
Age ≥60	58	30	28
Age <60	47	22	25
Female	34	16	18
Male	71	36	35
Smoking (no)	21	13	8
Smoking (yes)	84	39	45
Left lung	50	24	26
Right lung	55	28	27
Well and moderate	67	37	30
Poor	38	15	23
TNM (stages I and II)	56	40	16
TNM (stages III and IV)	49	12	37

## Data Availability

The data used to support the findings of this study are available from the corresponding author upon request.
